# The Practice of Medicine

**Published:** 1896-05

**Authors:** 


THE PRACTICE OF MEDICINE. A Condensed Manual for the Busy Practitioner. By Marvin A. Custis, M. D. Philadelphia: Bœricke & Tafel. 1896. Price, $2.00; by mail, $2.06.

This little work of 367 pages, in its elegant flexible morocco binding with gold edges, is very similar in external appearance to the *Accoucher's Emergency Manual*, by Dr. Yingling, published by the above-named firm in 1895. The author, in his preface, says: "In the description of the diseases the latest definite views of etiology and pathology have been given; and, in the treatment, only those remedies are mentioned that have a definite relation to the disease treated of.

"The symptoms of each remedy have been marked according to their estimated value.

"The standard works of both schools have been consulted in the preparation of this manual, and the indications for the remedies are those accepted by the homœopathic medical profession generally as trustworthy and accurate."

The above plan has been well carried out in this work. Almost every disease known to man is here treated in a few concise words. After the name of the disease its synonyms are given; then, in regular order, we find definition of the disease, etiology, pathology, symptoms, physical examination, duration, complications or sequelæ, prognosis, and, finally, the treatment, which comprises hygienic and dietetic as well as the medicinal treatment. The selection of remedies is good, as far as it is possible to group them under the name of a disease. The author has quoted liberally from the therapeutic experience of such physicians as Farrington, Hughes, Hale, Jahr, Bell, Lutze, Goodno, and a host of others, giving the reader an opportunity of knowing in what esteem a remedy was held in a certain disease by these well-known physicians.

The method in which a physician treats intermittent fever is generally an index of his treatment of other diseases, and of his position toward Homœopathy; so here does the treatment of intermittent fever somewhat index the character of the work before us, as shown in the following statement: "The quickest way to *check* intermittent fever is by giving a full dose, say ten to fifteen grains, of Quinine, about one hour before the expected chill; but, if you want to *cure* the patient, you must give the indicated remedy."

All in all, this is a work that will form for students a reliable compend on the Practice of Medicine, as also for the busy practitioner who wishes to carry with him on his visits to patients a work that comprises the whole scope of medicine in a condensed form. C. L. O.

